# Quantification of appetite-regulating hormones in children with hypothalamic and common obesity

**DOI:** 10.1210/jendso/bvag195

**Published:** 2026-08-19

**Authors:** Hoong-Wei Gan, Clare Leeson, Helen Aitkenhead, Helen Alexandra Spoudeas, Mehul Tulsidas Dattani

**Affiliations:** Genetics & Genomic Medicine Research & Training Department, UCL Great Ormond Street Institute of Child Health, London WC1N 1EH, UK; Department of Endocrinology, Great Ormond Street Hospital for Children NHS Foundation Trust, London WC1N 3JH, UK; Genetics & Genomic Medicine Research & Training Department, UCL Great Ormond Street Institute of Child Health, London WC1N 1EH, UK; Department of Chemical Pathology, Great Ormond Street Hospital for Children NHS Foundation Trust, London WC1N 3JH, UK; Department of Endocrinology, Great Ormond Street Hospital for Children NHS Foundation Trust, London WC1N 3JH, UK; Genetics & Genomic Medicine Research & Training Department, UCL Great Ormond Street Institute of Child Health, London WC1N 1EH, UK; Department of Endocrinology, Great Ormond Street Hospital for Children NHS Foundation Trust, London WC1N 3JH, UK

**Keywords:** hypothalamus, obesity, appetite regulation, hyperphagia, leptin, alpha-MSH

## Abstract

**Context:**

The pathophysiology of hypothalamic obesity (HyOb) remains incompletely understood with no effective treatments.

**Objective:**

We examined differences in appetite-regulating hormone concentrations between patients with HyOb, common obesity, and lean controls.

**Design:**

Multiway cross-sectional case-control study of patients aged 2 through 19 years.

**Setting:**

Two tertiary pediatric endocrinology centers.

**Patients:**

Cases were obese (body mass index [BMI] > +2 SD score [SDS], “HyOb”) and lean (“HyLean”) patients with congenital (septo-optic dysplasia) or acquired (suprasellar brain tumor) hypothalamic disorders. Controls had common obesity (“Ob”) or nonhypothalamic disorders and normal BMI (“Lean”).

**Main outcome measures:**

Relationships between the Dykens' Hyperphagia Questionnaire Score (DHQS), plasma or serum concentrations of leptin, insulin, α-melanocyte stimulating hormone (αMSH), brain-derived neurotrophic factor, oxytocin, acylated ghrelin, agouti-related peptide and copeptin, and BMI SDS.

**Results:**

Dykens' Hyperphagia Questionnaire Score did not differ between HyOb and Ob patients (24 [17-34] vs 24 [18-31]) but correlated with BMI SDS in patients with hypothalamic disorders (*P* = 0.02). HyOb and Ob patients exhibited similarly increased anorexigens (insulin, leptin) and decreased orexigens (ghrelin, agouti-related peptide) compared to HyLean and Lean patients. The rate of BMI increase was independently associated with lower αMSH (β = −0.23 [−0.36 to −0.11], *P* = .0007) and ghrelin (β=−0.004 [−0.01 to 0.00], *P* = .001) concentrations, suggesting that αMSH replacement may be a therapeutic target for HyOb. HyLean patients demonstrated intermediate insulin responses to glucose compared to other subcohorts.

**Conclusion:**

In our cohort, patients with HyOb appeared indistinguishable from Ob in terms of their appetite and appetite-regulating neuroendocrine circuitry. Higher αMSH concentrations are associated with reduced weight gain and may be a target for therapeutic intervention.

The hypothalamus plays a key role in the regulation of energy balance, and integrates endocrine signals from the adipose tissue, gastrointestinal tract, and other regions of the central nervous system to influence appetite, weight, and metabolism. These signals consist of both anorexigenic (eg, leptin, insulin, α-melanocyte-stimulating hormone [αMSH]) and orexigenic (eg, ghrelin, agouti-related peptide [AgRP]) hormones, which suppress and stimulate appetite respectively (1). More recently, hypothalamic hormones such as oxytocin (OXT) and vasopressin (AVP) have also been found to have anorexigenic effects (2-4). Hypothalamic obesity (HyOb) describes the weight gain observed either because of mutations in genes involved in these signalling pathways (monogenic obesity; eg, leptin deficiency), congenital maldevelopment of (eg, septo-optic dysplasia), or acquired damage to (eg, hypothalamo-pituitary tumors) this region. Although our understanding of this complex neuroendocrine circuitry continues to expand, it remains incomplete, and therefore the pathophysiology of HyOb is still not fully understood. Despite this, little observational research has been performed in humans to elucidate the physiological differences between patients with HyOb and common obesity. Contrastingly, various therapies have been trialed, including bariatric surgery, without a priori understanding of these underlying mechanisms, resulting in poor, short-term success rates with not insignificant side effects (1).

There have been few comparisons between the appetite-regulating endocrine milieu of HyOb and common obesity. These generally show that patients with HyOb exhibit disproportionate hyperleptinemia and hyperadiponectinemia (5, 6). These findings have been attributed to defective blood-brain barrier transport, receptor sensitivities, and post-receptor signalling. Lean subjects with hypothalamic damage also exhibit increased serum leptin concentrations despite normal body mass index (BMI) (6). Further differences in these neuroendocrine pathways have yet to be determined, and it remains unclear whether the pathogenesis of HyOb is distinguishable from common obesity. The rarity of patients with HyOb, and even more so patients with hypothalamic disorders but normal BMI, makes this particularly difficult to study. However, without this understanding, identification of novel therapeutic targets potentially amenable to treatment remains difficult.

We therefore aimed to clinically phenotype a cohort of obese and lean children with and without congenital and acquired hypothalamic structural disorders, determining the association between various features of the hypothalamic syndrome, focusing particularly on hyperphagia and HyOb, and the fasting plasma concentrations of a range of anorexigenic and orexigenic hormones involved in the hypothalamo-gut-adipose tissue circuitry.

## Materials and methods

### Recruitment of participants

Participants aged between 2 and 19 years were recruited from 2 tertiary pediatric endocrinology units in the United Kingdom, with a specialist interest in hypothalamo-pituitary disorders, between January 2014 and 2017. Cases (“HyOb”) were defined as obese children (BMI > 2 SD score [SDS] at baseline) with septo-optic dysplasia (SOD, “congenital”) or any hypothalamo-pituitary tumour surviving ≥6 months from end of treatment (“acquired”). Age- and BMI-matched controls either had common obesity (“Ob”, BMI > 2 SDS without a known cause), SOD or hypothalamic tumors with BMI ≤2 SDS (“HyLean”), or no known hypothalamic disorder and BMI ≤2 SDS (“Lean”). This lattermost group of patients had other nonhypothalamic, well-controlled endocrine diagnoses (eg, congenital adrenal hyperplasia [CAH], nonsuprasellar brain tumors that had not undergone radiotherapy). Patients were excluded if they had non-SOD- or tumor-related hypothalamic damage (eg, from radiotherapy exposure), or known genetic, syndromic, or other endocrine-related causes of obesity (eg, *LEP*, *LEPR*, *MC4R* mutations, Prader-Willi syndrome, poorly controlled CAH), inadequately treated hormone deficiencies or who were on insulin-sensitizing medications such as metformin.

### Clinical phenotyping

A retrospective clinical data review was performed and details regarding participants' demographics, auxology, pubertal stage, the presence of features of the hypothalamic syndrome (autism, learning difficulties, sleep, and temperature dysregulation), underlying diagnoses, endocrine, and previous oncological therapies were recorded. The Dykens' Hyperphagia Parental Questionnaire Score (DHQS) (7) was used to quantitatively assess the degree of hyperphagia. The number of hypothalamo-pituitary endocrine deficits present was quantified using the Endocrine Morbidity Score (EMS) (8). For a proportion of patients, auxological outcomes were also collected at 1-year follow-up to determine ΔBMI SDS and the rate of change in ΔBMI SDS.

### Sample collection

All participants underwent an oral glucose tolerance test (OGTT) as per departmental protocols. Briefly, patients were fasted overnight and admitted in the morning. An intravenous cannula was inserted before administering a 1.75-g/kg (maximum dose 75 g) oral glucose load. Blood samples were withdrawn at 0, 30, 60, 90, and 120 minutes for plasma glucose and serum insulin. Fasted samples were also drawn into chilled EDTA, lithium heparin (both containing 400 kIU/mL aprotinin) and plain serum tubes on ice for plasma/serum leptin, αMSH, OXT, brain-derived neurotrophic factor (BDNF), acylated ghrelin, AgRP, and copeptin (as a recognized surrogate marker for AVP secretion). For acylated ghrelin analysis, an aliquot of plasma was additionally acidified with 0.05 M HCl. All tubes were centrifuged at 1600 *g* for 15 minutes at 4 °C, following which the plasma supernatant was divided into aliquots and saved at −80 °C until analysis. Samples for plasma glucose analysis were collected into fluoride oxalate tubes and analyzed immediately.

### Hormone assays

Plasma insulin and copeptin were measured using widely available clinical immunoassays (Immulite 2000, Siemens Healthcare GmbH, Erlangen, Germany and B.R.A.H.M.S Kryptor, Thermo Fisher Scientific, Hennnigsdorf, Germany, respectively). Measurements of plasma OXT, αMSH, and acylated ghrelin were performed using internally validated enzyme immunoassays (9). Of note, plasma OXT was measured on unextracted samples because of our previously published concerns about the validity and consistency of solid phase extraction techniques (9). Serum leptin (range 0.1-95 ng/mL, interassay CV 3.9%-7.1%), plasma AgRP (range 5.5-1200 pg/mL, interassay CV 19.0%-29.6%), and BDNF (range 0.02-42.5 ng/mL, interassay CV 8.0%-10.1%) were measured by enzyme immunoassays validated by the University of Cambridge Institute of Metabolic Science, United Kingdom.

### Statistical analysis

Data were summarized as mean ± SD for parametric data, and median (IQR) for nonparametric data. Statistical analysis was performed using parametric and appropriately transformed nonparametric data, with statistical significance set at *P* < .05. For serum insulin, the Homeostatic Model of Assessment for Insulin Resistance (HOMA-IR) and Matsuda Insulin Sensitivity Index (Matsuda-ISI) were also calculated. Categorical variables were compared by the χ^2^-test, whereas continuous variables were compared by the Student *t*-test for independent samples, or by a 1-way ANOVA, with post hoc adjustments to make comparisons between individual subcohorts (Bonferroni and Tamhane T2 corrections where the Levene statistic *P* was >.05 and <.05, respectively). Correlations between continuous variables were explored by calculating the Pearson's *r* correlation coefficient. Regression coefficients with 95% CIs were estimated for BMI outcomes by univariate and multivariate linear regression. Predictors entered were the presence/absence of a hypothalamic lesion, age, baseline BMI SDS, Tanner stage, the presence/absence of autism, learning difficulties, sleep disturbance or temperature dysregulation, total DHQS, EMS, and the measured plasma/serum hormone concentrations. All statistical analyses were performed with SPSS version 25.0 (IBM, Armonk, New York, USA).

### Ethical approval

This study was approved by the London Bloomsbury National Research Ethics Committee on December 6, 2013 (REC reference 13/LO/1611). All participants and/or their parents or guardians provided informed consent.

## Results

### Clinical characteristics of patient and control populations (Table 1)

A total of 122 participants were included, of whom 34 had hypothalamo-pituitary tumors (9 craniopharyngiomas, 5 pilocytic astrocytomas, 5 pituitary adenomas [4 prolactinomas, 1 Cushing disease], 4 germinomas, 4 hamartomas, 3 unspecified thickenings of the pituitary stalk, 2 gangliogliomas, 1 pilomyxoid astrocytoma, and 1 lipoma/dermoid cyst). Of the Ob controls, 10 had concurrent nonhypothalamic endocrine diagnoses (8 had hypopituitarism but without hypothalamic abnormalities on magnetic resonance imaging, 1 had 21-hydroxylase-deficient CAH, and 1 had 45X/46XY gonadal dysgenesis). Of the Lean controls (n = 19), 15 had nonhypothalamic endocrine diagnoses (9 had 21-hydroxylase deficient CAH, 6 had hypopituitarism but without hypothalamic abnormalities on magnetic resonance imaging, and 1 had congenital adrenal hypoplasia).

**Table 1:** Baseline characteristics of patient cohort

Group	HyOb		HyLean		Ob	Lean	ANOVA/χ^2^-test *P* value
	SOD	Tumor	SOD	Tumor			
n	30	20	15	14	24	19	
Age at testing (years)	11.4 ± 4.4	13.0 ± 4.2	10.6 ± 3.5	12.0 ± 4.0	11.6 ± 2.8	9.2 ± 3.7	.07
%Female (n)	46.7% (14)	70.0% (14)	53.3% (8)	42.9% (6)	45.8% (11)	36.8% (7)	.44
%Non-White ethnicity (n)	23.3% (7)	50.0% (10)	20.0% (3)	50.0% (7)	33.3% (8)	31.6% (6)	.32
Age at diagnosis (years)	N/A	6.0 ± 5.5	N/A	9.5 ± 4.0	N/A	N/A	.05*^a^*
Time from diagnosis (years)	N/A	7.2 ± 5.1	N/A	2.8 ± 2.8	N/A	N/A	.00*^a^*
Height SDS at testing	−0.4 ± 1.4	−0.4 ± 1.8	−1.2 ± 1.2	−0.9 ± 0.9	0.4 ± 2.1	−0.6 ± 2.1	.10
Corrected height SDS at testing	−0.3 ± 1.5	−0.5 ± 1.6	−1.1 ± 1.5	−0.2 ± 1.5	0.6 ± 1.7	0.2 ± 1.7	.05
Weight SDS at testing	2.2 ± 1.2	2.1 ± 0.9	−0.5 ± 1.4	0.2 ± 0.8	2.4 ± 0.9	−0.3 ± 1.6	<.001
BMI SDS at testing	2.9 ± 0.8	2.6 ± 0.4	0.1 ± 1.7	0.9 ± 0.8	2.8 ± 0.5	0.1 ± 1.5	<.001
Tanner stage at testing	1 (1-4)	3 (2-5)	1 (1-3)	2 (1-5)	2 (1-4)	1 (1-3)	.08
Testicular volume at testing (mL, male patients only)	6.5 (2-15)	3 (2-4)	2 (1-12)	3 (2-10)	3.5 (2-6.5)	2 (2-2.5)	.11
%Surgery	3.3% (1)	75.0% (15)	0.0% (0)	50.0% (7)	0.0% (0)	0.0% (0)	.16*^a^*
%Radiotherapy	0.0% (0)	50.0% (10)	0.0% (0)	35.7% (5)	0.0% (0)	0.0% (0)	.50*^a^*
%Chemotherapy	0.0% (0)	40.0% (8)	0.0% (0)	14.3% (2)	4.2% (1)	5.3% (1)	.14*^a^*
%Autism (n)	43.3% (13)	5.0% (1)	60.0% (9)	7.1% (1)	4.2% (1)	5.3% (1)	<.001
%Learning difficulties (n)	66.7% (20)	50.0% (10)	80.0% (12)	28.6% (4)	16.7% (4)	21.1 % (4)	<.001
%Education, health and care plan (EHCP, n)*^b^*	63.3% (19)	45.0% (9)	80.0% (12)	35.7% (5)	12.5% (3)	10.5% (2)	<.001
%Special needs school (n)	46.7% (14)	25.0% (5)	66.7% (10)	14.3% (2)	8.3% (2)	5.3% (1)	<.001
%Sleep disturbance (n)	53.3% (16)	25.0% (5)	60.0% (9)	14.3% (2)	8.3% (2)	5.3% (1)	<.001
%Temperature dysregulation	0.0% (0)	0.0% (0)	6.7% (1)	0.0% (0)	4.2% (1)	10.5% (2)	.21
%GH deficiency	90.0% (27)	90.0% (18)	86.7% (13)	50.0% (7)	29.2% (7)	36.8% (7)	<.001
%TSH deficiency	80.0% (24)	65.0% (13)	66.7% (10)	50.0% (7)	20.8% (5)	26.3% (5)	<.001
%ACTH deficiency	80.0% (24)	50.0% (10)	73.3% (11)	35.7% (5)	20.8% (5)	15.8% (3)	<.001
%AVP deficiency	23.3% (7)	50.0% (10)	26.7% (4)	35.7% (5)	0.0% (0)	5.3% (1)	<.001
%LH/FSH deficiency	13.3% (4)	35.0% (7)	0.0% (0)	28.6% (4)	8.3% (2)	10.5% (2)	.06
%PRL deficiency	16.7% (5)	30.0% (6)	0.0% (0)	7.1% (1)	4.2% (1)	15.8% (3)	.09
%CPP	6.7% (2)	25.0% (5)	6.7% (1)	7.1% (1)	0.0% (0)	0.0% (0)	.01
EMS	3 ± 1	3 ± 2	2 ± 1	2 ± 2	1 ± 1	1 ± 2	<.001

Data are summarized as means ± SD for parametric data, and medians (IQR) for nonparametric data. Continuous variables were compared by the Student *t*-test or a 1-way ANOVA, whereas categorical variables were compared by the χ^2^ test (with Fisher's exact correlation for small values).

Abbreviations: AVP, vasopressin; BMI, body mass index; CPP, central precocious puberty; EMS, Endocrine Morbidity Score; PRL, prolactin; SOD, septo-optic dysplasia.

^*a*^Comparisons made only between tumor obese vs tumor lean subgroups.

^*b*^Patients with EHCPs require additional support for educational and/or physical needs at school.

Participants (49.2% female) were of a mean age of 11.3 ± 3.9 years with a median Tanner stage of 2 (1-4), with no significant differences between subgroups. Of the patients with hypothalamo-pituitary tumors, mean age at tumor diagnosis was 7.6 ± 5.1 years and the median time from diagnosis was 3.6 (1.8-7.6) years. In this subcohort, 67.6% had undergone surgical resection, 44.1% had received radiotherapy, and 35.3% had received chemotherapy, with no differences between groups. Patients with tumor-related HyOb had been followed-up for a longer period after diagnosis compared to those in the HyLean group (*P* = .004). The prevalence of autism was significantly higher in patients with SOD (51.2% vs 5.1%, *P* < .000001), whereas learning difficulties and sleep disturbances were more common in patients with any hypothalamic disorder (learning difficulties 85.2% vs 14.8%, *P* = .00003; sleep disturbances 40.5% vs 7.0%, *P* = .00009).

Mean EMS was significantly higher in patients with hypothalamic disorders (3 ± 2 vs 1 ± 2, *P* < .000001) because of an increased frequency of deficiencies in GH (82.3% vs 32.6%, *P* < .0001), TSH (68.4% vs 23.3%, *P* < .0001), ACTH (63.3% vs 18.6%, *P* < .0001), AVP (32.9% vs 2.3%, *P* < .0001), and central precocious puberty (11.4% vs 0.0%, *P* = .03), particularly in the HyOb subgroup. In patients on hormone supplementation, the mean GH, hydrocortisone, levothyroxine, and DDAVP doses were 0.7 ± 0.2 mg/m^2^/day, 10.5 ± 3.2 mg/m^2^/day, 1.7 ± 0.6 μg/kg/day, and 5.3 ± 3.9 μg/kg/day, respectively.

### Differences in appetite and food-related behavior

**Figure 1:**
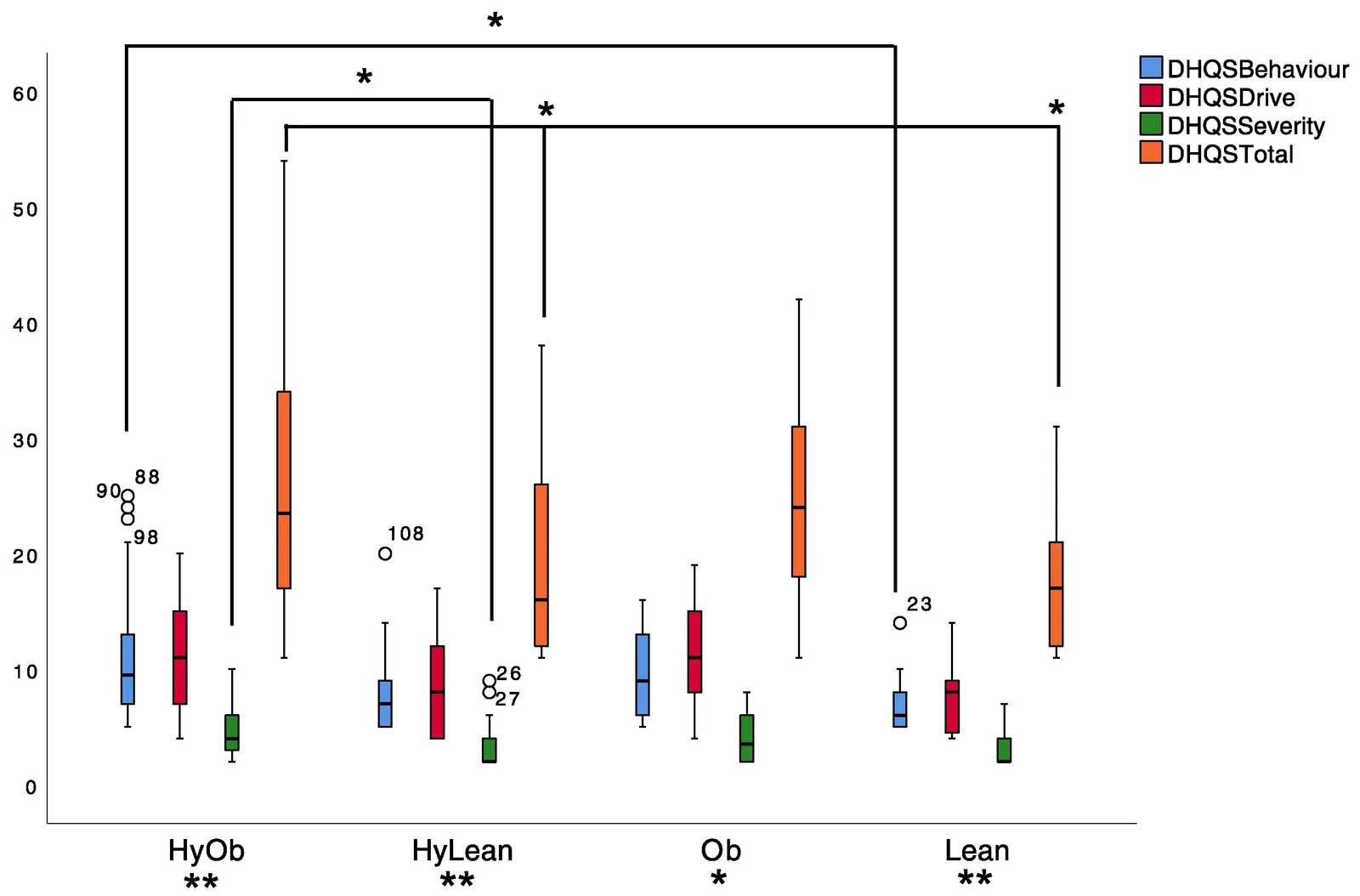
Comparison of Dykens Hyperphagia Questionnaire Scores (DHQS) and subscores for behavior, drive, and severity between the 4 different patient groups. Boxplots show median, IQR, and ranges for each variable. Data were transformed before analysis by 1-way ANOVA because of positive skewing. *P* values for the overall ANOVA are shown on the x-axis, and post hoc pairwise comparisons above the boxplots. *P < .05, **P < .01.

**Figure 2:**
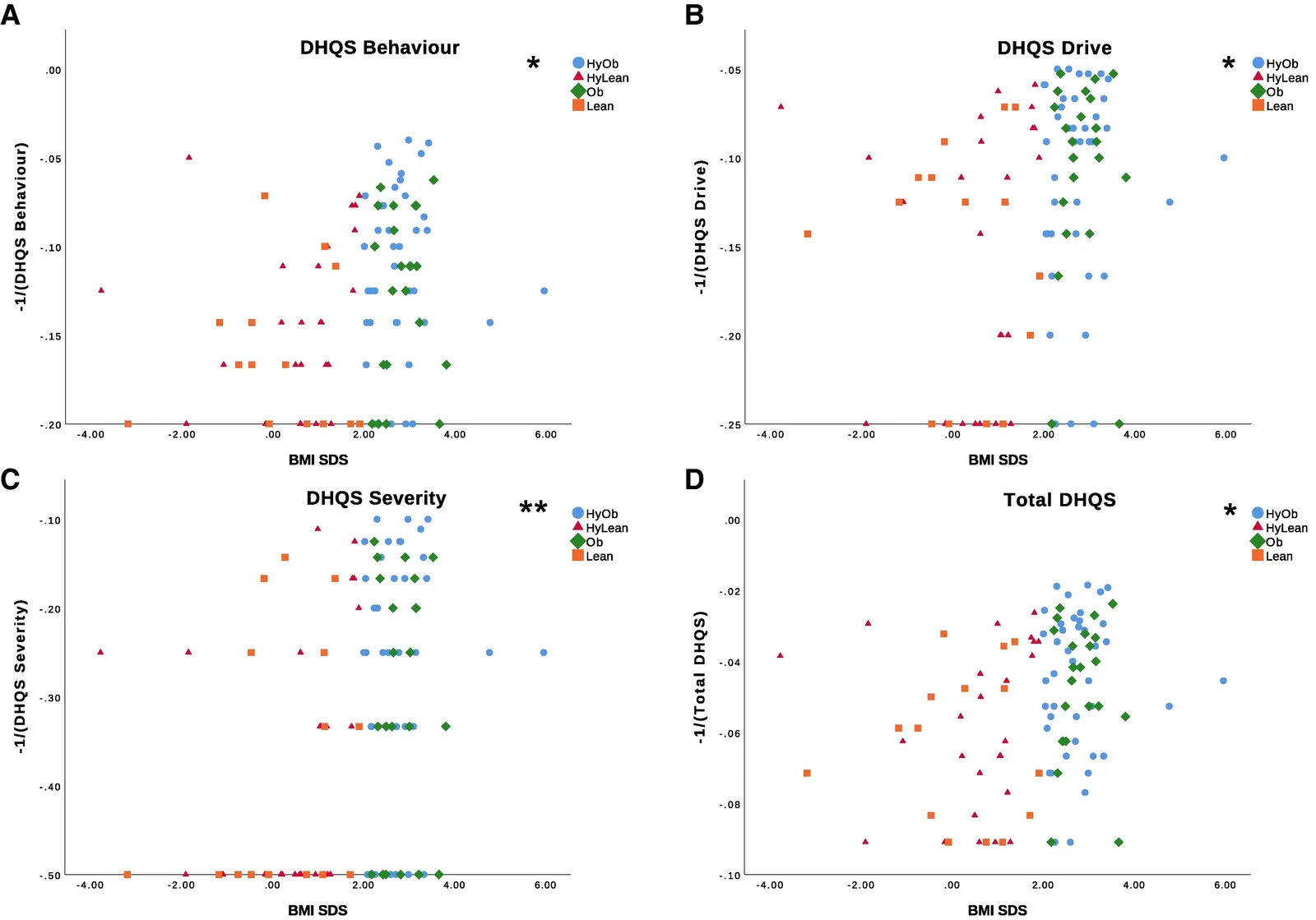
Correlation between the Dykens Hyperphagia Questionnaire Score (DHQS) and subscores for behavior, drive, and severity with BMI SDS in the four patient groups. Data for DHQS were transformed before Pearson's correlation coefficient analysis because of positive skewing. *P < .05, **P < .01.

DHQS data were available for 105/122 (86.1%) of participants (Fig. 1). Total DHQS and its subscores were significantly different between groups, with post hoc analyses showing that this was due to the HyOb group demonstrating significantly higher median total DHQS (24 [17-34]) compared to both HyLean (16 [12-26], *P* = .04) and Lean (17 [12-21], *P* = .03) groups, respectively. There were no significant differences between the HyOb and Ob groups in total DHQS or any of its subscores, and the only difference between HyOb and HyLean groups was in the Severity subscore (4 [3-6] vs 2 [2-4], *P* = .04). Total DHQS and its subscores all positively correlated with BMI SDS (Fig. 2), but this was only true for the group with hypothalamic lesions (HyOb and HyLean), regardless of BMI SDS (*R*^2^ Behavior = 0.27, *P* = .03; Drive = 0.27, *P* = .02; Severity = 0.34, *P* = .005; total DHQS = 0.29, *P* = .02).

### Correlation between appetite-regulating hormone concentrations and BMI

Because of difficulties with venous access, complete hormonal profiles were only obtained from 105 patients (42 HyOb, 25 HyLean, 23 Ob, 16 Lean), whereas a partial profile was obtained from the remaining 17 patients (10 HyOb, 2 Ob, 4 HyLean, 2 Lean).

#### Plasma glucose and serum insulin concentrations in response to oral glucose load

Fasting plasma glucose concentrations differed significantly between the 4 groups (HyOb 4.2 ± 0.4 mmol/L, HyLean 4.2 ± 0.6 mmol/L, Ob 4.3 ± 0.6 mmol/L, Lean 3.9 ± 0.5 mmol/L, *P* = .047), with no significant difference between the HyOb and HyLean groups (*P* = 1.0). Seven of 114 patients with available 2-hour plasma glucose concentrations were diagnosed with impaired glucose tolerance (2 HyOb, 1 HyLean, 3 Ob, 1 Lean), whereas 1 HyOb patient was diagnosed with type 2 diabetes (2-hour plasma glucose of 11.4 mmol/L).

**Figure 3:**
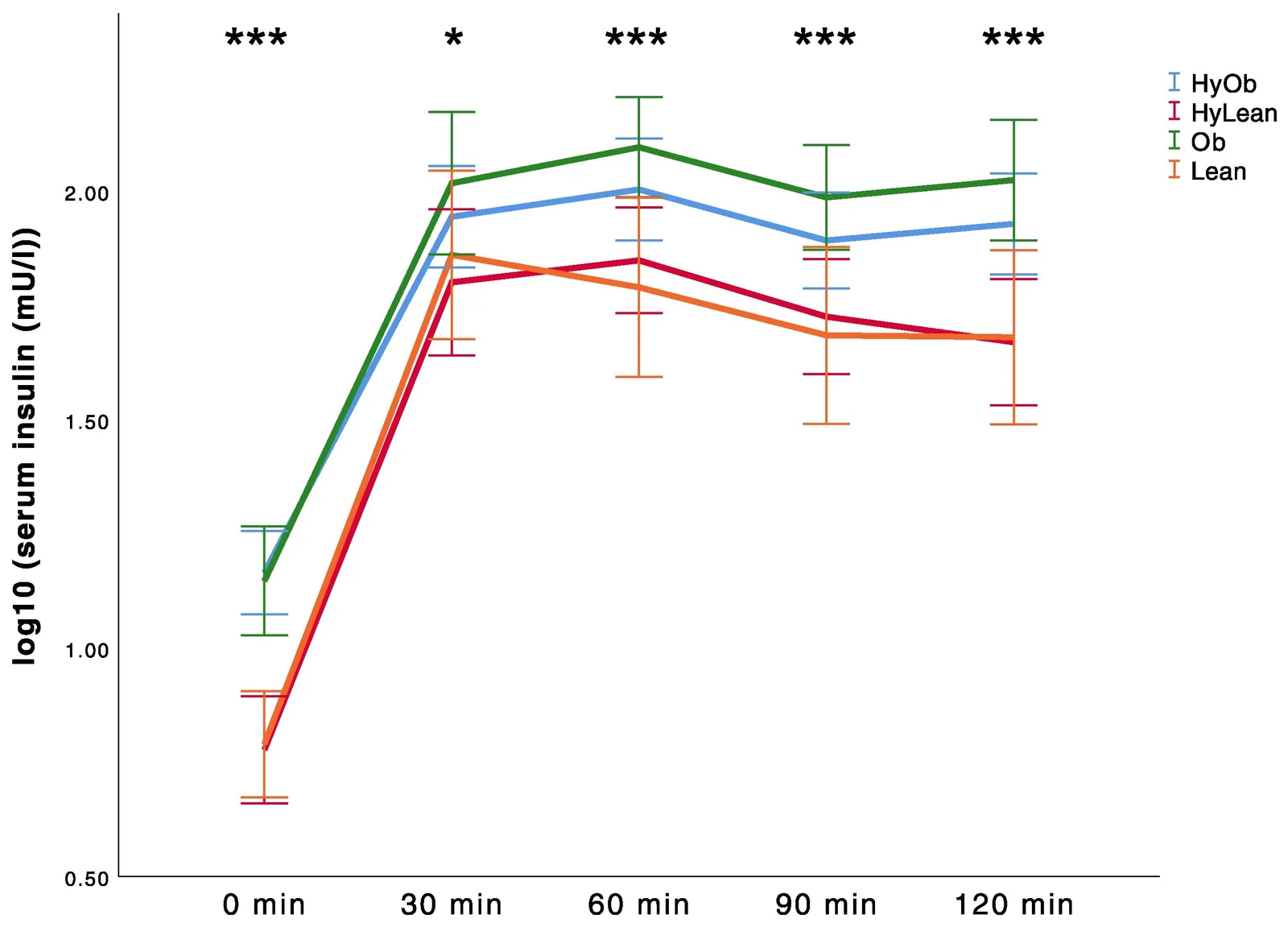
Serum insulin concentrations in response to a 1.75 g/kg (maximum 75 g) oral glucose load. Values shown are means ± 2 SE. Data were transformed before analysis by 1-way ANOVA. *P < .05, ***P < .001.

Serum insulin concentrations also differed between the groups throughout the OGTT (Fig. 3), with no significant differences between the HyOb and Ob groups, which together had higher fasting and 2-hour serum insulin concentrations, higher HOMA-IR, and lower Matsuda-ISI indices compared to the HyLean and Lean groups. In contrast, there were no significant differences in peak serum insulin or insulin area under the curve between HyOb and HyLean groups.

#### Fasting plasma and serum concentrations of other appetite-regulating hormones (Fig. 4)

Overall, peripheral concentrations of anorexigens (insulin, leptin) were increased, whereas peripheral (acylated ghrelin) and central (AgRP) orexigens were decreased in all forms of obesity. Similar to insulin, fasting serum leptin concentrations were not significantly different between HyOb and Ob groups (42.7 ± 24.6 vs 45.2 ± 23.0 ng/mL). Although there were no significant differences in plasma αMSH concentrations, there was a trend for Lean patients to have lower concentrations compared to the other groups (HyOb 2.0 ± 1.3 ng/mL, HyLean 2.2 ± 1.4 ng/mL, Ob 2.2 ± 1.4 ng/mL, Lean 1.5 ± 1.1 ng/mL, *P* = .5). Although there were no significant differences in plasma copeptin concentrations between BMI subgroups, patients with AVP deficiency (AVPD) unsurprisingly had lower concentrations (AVPD 1.4 [1.2-1.9] pmol/L vs no AVPD 4.5 [3.0-7.1] pmol/L, *P* < .000001). There were no significant differences between plasma BDNF and OXT concentrations between the subgroups.

**Figure 4:**
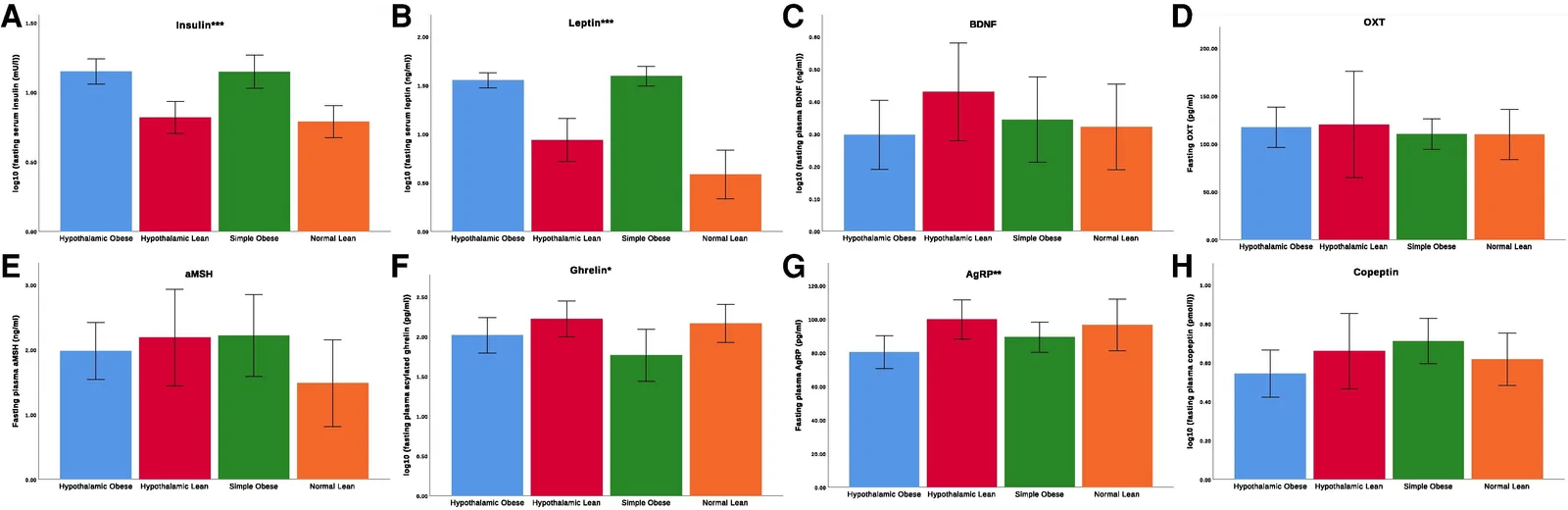
Plasma/serum concentrations of various appetite-regulating hormones in the 4 patient groups. Values shown are means ± 2 SE. Data for insulin, leptin, BDNF, ghrelin, and copeptin were transformed before analysis by 1-way ANOVA. Abbreviations: AgRP, Agouti-related peptide; BDNF, brain-derived neurotrophic factor; OXT, oxytocin; αMSH, α-melanocyte-stimulating hormone. *P < .05, **P < .01, ***P < .001.

### Correlation between appetite-regulating hormone concentrations and DHQS

Only plasma insulin and serum leptin concentrations showed correlations with DHQS (2-hour post-OGTT insulin *R*^2^ = 0.3, *P* = .02; HOMA-IR *R*^2^ = 0.3, *P* = .007; Matsuda-ISI *R*^2^ = −0.3, 0.0009; leptin *R*^2^ = 0.3, *P* = .005), with no other association found between DHQS or any of its subscores and the other appetite-regulating hormones measured.

### Predictors of change in BMI over time (Table 2)

Linear regression analyses were performed using data from 96 patients with sufficient follow-up duration to determine the factors which were associated with ΔBMI SDS and rate of ΔBMI SDS at the 1-year follow-up. Univariate analysis showed that a younger age at testing, a lower Tanner stage, and a lower BMI SDS at baseline were all predictive of a bigger change in BMI SDS at 1 year. Lower concentrations of peripheral anorexigen concentrations (leptin and insulin), as well as peripheral orexigen concentrations (acylated ghrelin) were negative predictors of ΔBMI SDS. On multivariate analysis of 51 patients with complete datasets (*R*^2^ = 0.52, F(17,33) = 2.092, *P* = .03), a higher EMS and lower αMSH were associated with a bigger change in BMI SDS, with a lower acylated ghrelin having a weaker independent association.

**Table 2:** Predictors of ΔBMI SDS and rate of ΔBMI SDS at 1 year follow-up

Independent variable	ΔBMI SDS				ΔBMI SDS/year			
	Univariate β (95% CI)	*P* value	Multivariate β (95% CI)	*P* value	Univariate β (95% CI)	*P* value	Multivariate β (95% CI)	*P* value
Hypothalamic lesion	−0.15 (−0.40 to 0.10)	.25	−0.32 (−0.74 to 0.11)	.14	−0.20 (−0.52 to 0.12)	.22	−0.31 (−0.79 to 0.17)	.15
Age at testing (years)	−0.04 (−0.07 to −0.01)	.01	−0.07 (−0.13 to 0.00)	.06	−0.05 (−0.09 to −0.01)	.01	−0.08 (−0.16 to 0.00)	.06
Tanner stage*^a^*	−0.49 (−0.92 to −0.06)	.03	0.09 (−0.80 to 0.97)	.84	−0.57 (−1.12 to −0.02)	.04	0.02 (−0.93 to 0.98)	.78
BMI SDS at testing	−0.17 (−0.26 to −0.09)	<.001	0.06 (−0.18 to 0.30)	.62	−0.23 (−0.34 to −0.13)	<.001	−0.02 (−0.26 to 0.23)	.78
Autism	−0.09 (−0.41 to 0.21)	.53	0.06 (−0.43 to 0.55)	.79	−0.08 (−0.47 to 0.32)	.70	0.19 (−0.33 to 0.7)	.67
Learning difficulties	−0.07 (−0.32 to 0.18)	.58	−0.37 (−0.79 to 0.06)	.09	−0.14 (−0.46 to 0.18)	.38	−0.43 (−0.86 to 0.01)	.09
Sleep disturbances	−0.16 (−0.43 to 0.11)	.24	−0.21 (−0.66 to 0.24)	.35	−0.24 (−0.58 to 0.11)	.17	−0.31 (−0.77 to 0.15)	.24
Temperature dysregulation	0.05 (−0.65 to 0.76)	.89	Not included*^b^*	N/A	0.22 (−0.68 to 1.13)	.63	Not included*^b^*	N/A
Total DHQS*^a^*	−2.31 (−8.14 to 3.53)	.43	0.53 (−7.46 to 8.51)	.89	−4.66 (−11.50 to 2.19)	.18	−1.76 (−10.47 to 6.95)	.82
EMS	0.002 (−0.6 to 0.07)	.95	0.22 (0.09 to 0.36)	.00	0.01 (−0.7 to 0.10)	.79	−0.002 (−0.01 to 0.00)	.00
Fasting insulin (mU/L)*^a^*	−0.49 (−0.80 to −0.17)	.00	−0.26 (−0.81 to 0.30)	.35	−0.52 (−0.88 to −0.15)	.01	−0.29 (−0.83 to 0.24)	.29
Fasting OXT (pg/mL)	0.00 (0.00-0.00)	.51	−0.002 (−0.01 to 0.00)	.08	0.00 (0.00-0.00)	.59	0.00 (−0.01 to 0.00)	.08
Fasting leptin (ng/mL)*^a^*	−0.36 (−0.59 to −0.14)	.00	−0.17 (−0.78 to 0.44)	.57	−0.49 (−0.79 to −0.20)	.00	0.06 (−0.58 to 0.70)	.97
Fasting αMSH (ng/mL)	−0.06 (−0.17 to 0.06)	.30	−0.18 (−0.32 to −0.05)	.01	−0.15 (−0.27 to −0.02)	.03	−0.23 (−0.36 to 0.11)	.00
Fasting BDNF (ng/mL)*^a^*	0.05 (−0.34 to 0.45)	.78	−0.09 (−0.57 to 0.38)	.69	0.09 (−0.42 to 0.60)	.73	0.03 (−0.50 to 0.55)	.99
Fasting acylated ghrelin (pg/mL)	−0.04 (−0.79 to −0.02)	.04	−0.004 (−0.01 to 0.00)	<.001	−0.001 (0.00-0.00)	.14	−0.004 (−0.01 to 0.00)	.00
Fasting AgRP (pg/mL)	0.00 (−0.003 to 0.01)	.45	0.01 (0.00-0.02)	.05	0.00 (0.00-0.01)	.29	0.01 (0.00-0.01)	.03
Fasting copeptin (pmol/L)*^a^*	−0.05 (−0.53 to 0.44)	.84	−0.01 (−0.65 to 0.64)	.98	−0.14 (−0.78 to 0.51)	.68	−0.01 (−0.05 to 0.03)	1.00

Abbreviations: AgRP, Agouti-related peptide; BDNF, brain-derived neurotrophic factor; BMI, body mass index; DHQS, Dykens Hyperphagia Questionnaire Score; EMS, Endocrine Morbidity Score; OXT, oxytocin; αMSH, α-melanocyte stimulating hormone.

^*a*^Data for Tanner stage, DHQS, HOMA-IR, fasting leptin, BDNF, and copeptin concentrations were positive skewed and transformed before regression analyses.

^*b*^Temperature dysregulation was not included in the final multivariate model as it was not present in the final 51 patients used for multivariate analysis.

Because of the slight variation in the duration of follow-up between patients (0.9 ± 0.2 years), the rate of change in BMI SDS was also analyzed as a dependent variable. Similar to the analysis for ΔBMI SDS, a younger age at testing, a lower Tanner stage, and a lower baseline BMI SDS were all associated with a greater rate of BMI SDS gain at 1 year. Lower peripheral (leptin and insulin) and central (αMSH) concentrations were associated with more rapid BMI SDS gain, with no significant effect from the orexigens. Multivariate analysis of 55 patients (*R*^2^ = 0.585, F(17,37) = 3.066, *P* = .002) showed that a younger age at testing and lower fasting αMSH were independently associated with a greater rate of BMI SDS gain, with a lower acylated ghrelin, and a higher fasting AgRP having weaker independent associations.

## Discussion

This is the largest study comparing the clinical features, food-directed behavior, and appetite-regulating endocrine physiology in a cohort of children with congenital and acquired hypothalamic structural disorders. We found that even in common obesity, some features of hyperphagia classically attributed to HyOb were also present, as seen in the similar DHQS between these groups. However, the DHQS was more predictive of BMI in patients with hypothalamic disorders. This is in keeping with the original intention of DHQS for use in Prader-Willi syndrome, where a similar correlation was found (7). Therefore, its usefulness lies not in the diagnosis of hyperphagia but its severity in patients with hypothalamic dysfunction.

The concentration of all appetite-regulating hormones studied did not appear to differ between the HyOb and Ob groups, with both exhibiting appropriate compensatory responses of increased peripheral anorexigens (leptin, insulin) and decreased peripheral (ghrelin) and central (AgRP) orexigens. These hormones were also positively and negatively correlated with BMI SDS, respectively. Similar to the relationship with BMI, the degree of hyperphagia observed was unsurprisingly positively correlated with serum leptin and insulin and negatively correlated with plasma acylated ghrelin. These findings suggest that these homeostatic pathways governing weight and appetite in both groups of obese patients remain intact, in keeping with previous literature (5, 6, 10), in contrast to Prader-Willi syndrome, the archetypal genetic HyOb disorder where plasma ghrelin concentrations are inappropriately increased (11).

Although AgRP is largely synthesized centrally by the arcuate nucleus, peripheral plasma AgRP concentrations were also shown to be negatively correlated with BMI SDS. Previous studies have shown that peripheral AgRP concentrations increase in response to caloric restriction or fasting and decrease in response to refeeding (12, 13). Contrastingly, studies on the association between plasma AgRP and BMI have been more mixed—1 study in children showed no significant correlation (14), whereas other studies in adults showed both negative (15) and positive (16) relationships. These findings suggest that AgRP is likely a transient marker of change in energy status (fasting vs fed), rather than of long-term weight gain or loss. In our cohort, AgRP samples were always obtained in a fasted state, and as such may have shown a stronger correlation with BMI SDS than it would have done in a fed state.

Of the other appetite-regulating hormones studied, no correlation could be found between their plasma concentrations and BMI SDS. Indeed, the published evidence documenting the association between plasma OXT, αMSH, and BDNF with BMI is inconclusive. The association between plasma OXT concentrations and BMI is variable (17, 18), but this is the first study to report a complete lack of association. In a subcohort of patients, we measured plasma OXT concentrations in response to the OGTT and similarly demonstrated no response (data not shown). This is in contrast to previous studies that have demonstrated that glucose can stimulate hypothalamic *Oxt* expression and increase endogenous plasma OXT concentrations (19), as well as other studies suggesting an anorexigenic effect of exogenous OXT on appetite and weight (2, 3).

Similarly, previous studies have shown variable associations between plasma αMSH and BDNF concentrations with BMI (14, 16, 20, 21). Caloric restriction in particular has been shown to increase plasma αMSH concentrations in adolescents (22). Notably, our data for plasma copeptin are not concordant with findings by other authors, who have consistently described a positive correlation with BMI, the metabolic syndrome, and increased cardiovascular risk (23, 24). Other authors have found that copeptin increases further postbariatric surgery, correlating with the degree of weight loss (25). This lack of correlation may be due to the high numbers of patients with AVPD in this preselected cohort with hypothalamic structural abnormalities (>20%), who have significantly lower plasma copeptin concentrations, compared to the general population. Indeed, we did not find a correlation between the presence of AVPD and BMI SDS in our cohort.

We also examined which variables determined the BMI trajectory of a subcohort of these patients over time by regression analyses. Although ΔBMS SDS was unsurprisingly independently associated with EMS and inversely associated with acylated ghrelin concentrations as markers respectively of increased hypothalamo-pituitary dysfunction and increased compensation for a net positive energy balance, the negative association with the anorexigen αMSH suggests that this may be a factor contributing to increasing weight over time. A similar association was found using the rate of ΔBMI SDS change over time as an outcome, with additional weaker negative associations with age at testing (possibly due to a lead time bias resulting from patients with congenital HyOb) and plasma AgRP concentrations. The association with αMSH is relevant as the MC4R agonist setmelanotide is already licensed for treating LEPR (26), POMC (27), and PCSK1 (28) deficiencies. Indeed, a recently published phase 3 trial has shown excellent results of its use in acquired HyOb (29).

This study is limited by the methods used to quantify hyperphagia and the secretion of various appetite-regulating hormones. To date, the DHQS has only been validated for use in Prader-Willi syndrome (7), and we did not use other questionnaires such as the Children's Eating Behavior Questionnaire (30) and the Three-Factor Eating Questionnaire (31). Contrastingly, ad libitum food intake studies, which are regarded as the gold standard, are not logistically or ethically feasible in a cohort of children with autism, learning difficulties, and visual impairment, all of which would have rendered them inappropriate.

The clinical significance of plasma measurements of centrally secreted hormones is also debatable. Some are secreted near areas where the blood-brain barrier is naturally incomplete (area postrema, median eminence) such as the nucleus tractus solitarius (αMSH), ARC (αMSH and AgRP), and posterior pituitary (OXT), suggesting plasma hormone concentrations may reflect central nervous system secretion. However, previous literature examining the correlation between plasma and cerebrospinal fluid concentrations of these hormones is inconsistent (15, 32, 33). Ideally, measurement of the cerebrospinal fluid appetite-regulating hormone milieu would provide a better picture of the dysregulation leading to obesity, but this would be difficult to perform in this cohort of children. Clinically validated immunoassays for the measurement of hormones such as OXT also do not yet exist. We have previously internally validated the OXT assay used in this paper, and in the process of doing so found issues with some widely available immunoassays, particularly with regard to the often recommended preanalytical extraction process and the specificity of antibodies used (9). As a result, we only analyzed unextracted plasma samples using an immunoassay that used a monoclonal secondary antibody. More recently, immunoassays for neurophysin I, the carrier protein for OXT, have also become available (34), but accurate assays for neurophysin I were not available at the time of this study. Additionally, we did not measure other known appetite-regulating hormones such as cholecystokinin and neuropeptide Y.

The Ob and Lean controls used in this study were also not healthy children. This was due to the intensity of the OGTT, requiring a period of fasting, insertion of a cannula, and multiple blood draws. Some of these patients (6 Ob, 1 Lean), although not having structural abnormalities directly involving the hypothalamus, also had an ectopic posterior pituitary ± an absent pituitary stalk, which could be regarded as a hypothalamic disorder (35). All other controls had other nonhypothalamic diagnoses, including well-controlled CAH. Although the hydrocortisone doses being used to treat these patients were within the usual therapeutic range (5.8-20.2 mg/m^2^/day), it is possible that glucocorticoid replacement could have impacted the hypothalamic-gut circuitry. Last, 1 patient had 45X/46XY gonadal dysgenesis and could have had differences in their sex steroid concentrations that may also have influenced the concentration of appetite-regulating hormones. Together, these factors could have reduced the likelihood of detecting differences between the HyOb, HyLean, Ob, and Lean groups.

Additionally, of the 122 patients recruited to the study, auxological follow-up data were only available for 96 patients, and only at 1 year. Further research is required to determine what factors predict changes in weight and BMI over a longer period. As part of this, the BMI trajectory of HyLean patients remains to be determined, particularly as they exhibited similar peak serum insulin concentrations to the HyOb group in response to an OGTT, with a trend for higher BDNF and AgRP concentrations compared to the other subgroups. These findings suggest that HyLean patients may exhibit early dysregulation of their hypothalamic-gut circuitry. Lastly, the responses to an oral glucose load were only examined for insulin and OXT, and responses of a vast number of other appetite-regulating hormones including NPY, CCK, nesfatin, and GLP-1 were not determined.

In summary, we present data describing and correlating, for the first time, the pattern of eating behaviors and appetite-regulating hormone concentrations in patients with congenital and acquired hypothalamic structural damage, in comparison to their obese and lean counterparts without these disorders. Given the accruing number of patients with congenital and acquired HyOb thanks to better endocrine and oncological care, it is crucial that we understand its pathophysiology to derive more effective medical therapeutic options in a pediatric cohort where bariatric surgery would be considered invasive and potentially risky in the presence of other hormone deficits. Furthermore, the similarity between the HyOb and Ob subgroups supports the notion that understanding the pathophysiology of HyOb is crucial to managing the increasing prevalence of childhood obesity in the general population.

## Data Availability

Some or all datasets generated during and/or analyzed during the current study are not publicly available but are available from the corresponding author on reasonable request. Requests for fully anonymized data should be submitted to the corresponding author for consideration up to 5 years from publication, with an accompanying research proposal which has undergone appropriate ethical approval.

## Abbreviations

AgRP: agouti-related peptide
AVP: vasopressin
AVPD: vasopressin deficiency
BDNF: brain-derived neurotrophic factor
BMI: body mass index
CAH: congenital adrenal hyperplasia
DHQS: Dykens Hyperphagia Questionnaire Score
EMS: Endocrine Morbidity Score
HOMA-IR: Homeostatic Model of Assessment for Insulin Resistance
HyOb: hypothalamic obesity
OGTT: oral glucose tolerance test
OXT: oxytocin
SDS: SD score
SOD: septo-optic dysplasia
αMSH: α-melanocyte stimulating hormone
